# Supplementary material for: Comparative genomics provides new insights into the diversity, physiology, and sexuality of the only industrially exploited tremellomycete: Phaffia rhodozyma
Source: BMC Genomics. 2016 Nov 9;17:901. doi: 10.1186/s12864-016-3244-7 (PMC5103461; doi:10.1186/s12864-016-3244-7)
Supplement: Additional file 6: — List of orphan genes with links to PFAM (related to Additional file 1: Table S1). (ZIP 1428 kb) [file 12864_2016_3244_MOESM6_ESM.zip › BLAST_HTML_FTR/G01756_P.html]

BLAST Search Results


```
BLASTP 2.2.27+


Reference:
Stephen F. Altschul, Thomas L. Madden, Alejandro A. Schäffer,
Jinghui Zhang, Zheng Zhang, Webb Miller, and David J. Lipman (1997),
"Gapped BLAST and PSI-BLAST: a new generation of protein database
search programs", Nucleic Acids Res. 25:3389-3402.


Reference for
composition-based statistics:
Alejandro A. Schäffer, L. Aravind, Thomas L. Madden, Sergei
Shavirin, John L. Spouge, Yuri I. Wolf, Eugene V. Koonin, and
Stephen F. Altschul (2001), "Improving the accuracy of PSI-BLAST
protein database searches with composition-based statistics and
other refinements", Nucleic Acids Res. 29:2994-3005.


Database: nr
           71,551,133 sequences; 26,053,659,533 total letters


Query= G01756_P

Length=246
                                                                      Score     E
Sequences producing significant alignments:                          (Bits)  Value

emb|CDZ96956.1|  hypothetical protein [Xanthophyllomyces dendrorh...   254    2e-82
emb|CDZ96955.1|  hypothetical protein [Xanthophyllomyces dendrorh...   122    1e-31
gb|KOG96895.1|  WSC2-like protein [Saccharomyces eubayanus]           48.5    0.003
ref|XP_003038930.1|  expressed protein [Schizophyllum commune H4-...  47.4    0.006
emb|CEG82074.1|  hypothetical protein RMATCC62417_16198 [Rhizopus...  45.4    0.027
gb|ELQ33413.1|  hypothetical protein OOU_Y34scaffold00946g7 [Magn...  45.1    0.042
gb|KND86374.1|  hypothetical protein TOPH_09001 [Tolypocladium op...  45.1    0.044
ref|XP_003717394.1|  hypothetical protein MGG_10115 [Magnaporthe ...  45.1    0.044
emb|CRJ79720.1|  hypothetical protein BN1708_000082, partial [Ver...  45.1    0.051
ref|XP_009656719.1|  hypothetical protein VDAG_09463 [Verticilliu...  45.1    0.054
emb|CEJ00600.1|  hypothetical protein RMCBS344292_14652 [Rhizopus...  43.9    0.075
ref|XP_008023814.1|  hypothetical protein SETTUDRAFT_107372 [Seto...  43.9    0.10 
gb|EJS42050.1|  wsc2p [Saccharomyces arboricola H-6]                  43.5    0.14 
gb|EJT96809.1|  hypothetical protein DACRYDRAFT_112426 [Dacryopin...  43.5    0.14 
gb|KIJ96155.1|  hypothetical protein K443DRAFT_682489 [Laccaria a...  42.4    0.28 
emb|CEG64853.1|  hypothetical protein RMATCC62417_01760 [Rhizopus...  42.0    0.31 
ref|XP_008037507.1|  hypothetical protein TRAVEDRAFT_47227 [Trame...  42.4    0.33 
emb|CEI97351.1|  hypothetical protein RMCBS344292_11486 [Rhizopus...  42.0    0.34 
emb|CEG64852.1|  hypothetical protein RMATCC62417_01760 [Rhizopus...  42.0    0.36 
ref|XP_002552621.1|  KLTH0C09196p [Lachancea thermotolerans] >emb...  42.0    0.43 
gb|KIM94625.1|  hypothetical protein OIDMADRAFT_34638 [Oidiodendr...  41.6    0.47 
gb|KIV87214.1|  hypothetical protein PV11_02776 [Exophiala sideris]   41.6    0.51 
gb|KIK53882.1|  hypothetical protein GYMLUDRAFT_49178 [Gymnopus l...  41.6    0.53 
ref|XP_003666942.1|  hypothetical protein MYCTH_2312114 [Myceliop...  41.6    0.56 
ref|XP_001267184.1|  hypothetical protein NFIA_107790 [Neosartory...  41.6    0.56 
ref|XP_005996350.1|  PREDICTED: ephrin type-A receptor 2 [Latimer...  41.6    0.69 
gb|KFH43510.1|  hypothetical protein ACRE_057260 [Acremonium chry...  41.2    0.69 
emb|CCA70236.1|  hypothetical protein PIIN_04175 [Piriformospora ...  41.2    0.70 
gb|KFY41578.1|  hypothetical protein V494_02916 [Pseudogymnoascus...  41.2    0.81 
ref|XP_006453939.1|  hypothetical protein AGABI2DRAFT_175677 [Aga...  40.8    0.86 
gb|KIK95982.1|  hypothetical protein PAXRUDRAFT_139503 [Paxillus ...  40.4    1.2  
ref|XP_003030568.1|  expressed protein [Schizophyllum commune H4-...  39.7    1.3  
gb|KIM55198.1|  hypothetical protein SCLCIDRAFT_1221343 [Sclerode...  40.4    1.3  
dbj|GAO83721.1|  mediator of RNA polymerase II transcription subu...  40.4    1.3  
ref|XP_011123584.1|  hypothetical protein AOL_s00083g103 [Arthrob...  40.0    1.5  
gb|KNE72351.1|  hypothetical protein AMAG_16835 [Allomyces macrog...  39.7    2.3  
ref|XP_008726262.1|  hypothetical protein G647_03695 [Cladophialo...  39.3    2.9  
ref|WP_049673322.1|  hypothetical protein [Desulfocarbo indianens...  39.3    3.5  
ref|XP_010869136.1|  PREDICTED: transmembrane protein 248 isoform...  38.9    3.8  
ref|XP_010869137.1|  PREDICTED: transmembrane protein 248 isoform...  38.9    4.0  
emb|CBY12936.1|  unnamed protein product [Oikopleura dioica]          38.9    4.1  
gb|EKG09317.1|  hypothetical protein MPH_13631 [Macrophomina phas...  38.9    4.1  
emb|CBY43440.1|  unnamed protein product [Oikopleura dioica]          38.5    4.6  
gb|KIK55984.1|  hypothetical protein GYMLUDRAFT_248203 [Gymnopus ...  38.5    4.7  
gb|KIL85067.1|  hypothetical protein FAVG1_11495 [Fusarium avenac...  38.1    4.7  
gb|KIY49433.1|  hypothetical protein FISHEDRAFT_72756 [Fistulina ...  38.5    5.5  
gb|EQL31746.1|  hypothetical protein, variant 3 [Blastomyces derm...  37.7    7.2  
ref|XP_007775861.1|  hypothetical protein CONPUDRAFT_86099 [Conio...  38.1    7.2  
ref|XP_001796011.1|  hypothetical protein SNOG_05608 [Phaeosphaer...  37.4    7.3  
gb|KMW67547.1|  ER membrane protein Wsc4, variant 4 [Blastomyces ...  37.4    7.7  
gb|KJA24276.1|  hypothetical protein HYPSUDRAFT_136642 [Hypholoma...  38.1    7.7  


 >emb|CDZ96956.1| hypothetical protein [Xanthophyllomyces dendrorhous]
Length=129

 Score =  254 bits (650),  Expect = 2e-82, Method: Compositional matrix adjust.
 Identities = 129/129 (100%), Positives = 129/129 (100%), Gaps = 0/129 (0%)

Query  117  MFMVPAQASVFGTKSAEVNGEKTLRSIETGGHARTPSEVPLLTEPPLAPMPYSVPPISTM  176
            MFMVPAQASVFGTKSAEVNGEKTLRSIETGGHARTPSEVPLLTEPPLAPMPYSVPPISTM
Sbjct  1    MFMVPAQASVFGTKSAEVNGEKTLRSIETGGHARTPSEVPLLTEPPLAPMPYSVPPISTM  60

Query  177  APARLIQGSVSLSSLRPSRSEQSIYPLLSSSASSDELMQAHDPFGAYAPVSIIPPRSIPR  236
            APARLIQGSVSLSSLRPSRSEQSIYPLLSSSASSDELMQAHDPFGAYAPVSIIPPRSIPR
Sbjct  61   APARLIQGSVSLSSLRPSRSEQSIYPLLSSSASSDELMQAHDPFGAYAPVSIIPPRSIPR  120

Query  237  SSSRTTLGF  245
            SSSRTTLGF
Sbjct  121  SSSRTTLGF  129


>emb|CDZ96955.1| hypothetical protein [Xanthophyllomyces dendrorhous]
Length=64

 Score =  122 bits (305),  Expect = 1e-31, Method: Compositional matrix adjust.
 Identities = 64/64 (100%), Positives = 64/64 (100%), Gaps = 0/64 (0%)

Query  1   MISTEELKRQQAGLDAEGSMSTAEGRRTSTYVIYSTSTLAETIEPSSTSTLLSSSSSHAS  60
           MISTEELKRQQAGLDAEGSMSTAEGRRTSTYVIYSTSTLAETIEPSSTSTLLSSSSSHAS
Sbjct  1   MISTEELKRQQAGLDAEGSMSTAEGRRTSTYVIYSTSTLAETIEPSSTSTLLSSSSSHAS  60

Query  61  PRSM  64
           PRSM
Sbjct  61  PRSM  64


>gb|KOG96895.1| WSC2-like protein [Saccharomyces eubayanus]
Length=486

 Score = 48.5 bits (114),  Expect = 0.003, Method: Compositional matrix adjust.
 Identities = 24/67 (36%), Positives = 41/67 (61%), Gaps = 2/67 (3%)

Query  52   LSSSSSHASPRSMSAAAIAGGVLGGIGFTLICSIILALFFRSRRRRQASRELPLQDNKHA  111
            L   SS +  + +S  AIAG V+G +  T+I + ++ L+F  ++RRQ S+++ L++ KH 
Sbjct  289  LDGKSSTSKSKHLSGGAIAGIVVGVVCGTVILAALVILYFVWKKRRQTSQQVDLEETKH-  347

Query  112  AYHPEMF  118
             Y P  F
Sbjct  348  -YQPYSF  353


>ref|XP_003038930.1| expressed protein [Schizophyllum commune H4-8]
 gb|EFJ04028.1| expressed protein [Schizophyllum commune H4-8]
Length=362

 Score = 47.4 bits (111),  Expect = 0.006, Method: Compositional matrix adjust.
 Identities = 27/71 (38%), Positives = 39/71 (55%), Gaps = 6/71 (8%)

Query  54   SSSSHASPRSMSAAAIAGGVLGGIGFTLICSIILALFFRSRRRRQASRELPLQDNKHAAY  113
            ++++ A+ +S  A  IAG V+G I F  I S +L  FFR RRR  A      + ++    
Sbjct  173  TNTAAAAGKSNDAGVIAGSVVGSIAFLAIGSALLWWFFRGRRRTSA------RGSESGIL  226

Query  114  HPEMFMVPAQA  124
            HPE F+ P QA
Sbjct  227  HPEPFVQPPQA  237


>emb|CEG82074.1| hypothetical protein RMATCC62417_16198 [Rhizopus microsporus]
Length=296

 Score = 45.4 bits (106),  Expect = 0.027, Method: Compositional matrix adjust.
 Identities = 25/75 (33%), Positives = 44/75 (59%), Gaps = 6/75 (8%)

Query  28   TSTYVIYSTSTLAETIEPSSTSTLLSSSSSHASPRSMSAAAIAGGVLGGIGFTLICSIIL  87
            TST+ + STS    T      +++ +  S+ A P S++++AIAGGV+GG+ F ++  II 
Sbjct  145  TSTFTVVSTSYSMYT------TSVPTDISNTAQPSSVNSSAIAGGVVGGVAFIVLIGIIG  198

Query  88   ALFFRSRRRRQASRE  102
                R R  ++ +R+
Sbjct  199  FFVLRKRNPKKTTRD  213


>gb|ELQ33413.1| hypothetical protein OOU_Y34scaffold00946g7 [Magnaporthe oryzae 
Y34]
 gb|ELQ69438.1| hypothetical protein OOW_P131scaffold00154g4 [Magnaporthe oryzae 
P131]
Length=587

 Score = 45.1 bits (105),  Expect = 0.042, Method: Compositional matrix adjust.
 Identities = 34/91 (37%), Positives = 46/91 (51%), Gaps = 4/91 (4%)

Query  19   SMSTAEGRRTSTYVIYSTSTLAETIEPSSTSTLLSSSSSHASPRSMSAAAIAGGVLGGIG  78
            + S A G      V   T TL   I PSST T  S++S      S SAAA AG   G IG
Sbjct  149  TQSPASGTDAPLAVAKDTPTLP--IAPSSTPTPTSAASKAED--SGSAAAKAGIAFGIIG  204

Query  79   FTLICSIILALFFRSRRRRQASRELPLQDNK  109
              L+ S+++   F+ R+++   REL  +D K
Sbjct  205  GLLVISLLIFFLFKKRKQQMERRELQAEDEK  235


>gb|KND86374.1| hypothetical protein TOPH_09001 [Tolypocladium ophioglossoides 
CBS 100239]
Length=413

 Score = 45.1 bits (105),  Expect = 0.044, Method: Compositional matrix adjust.
 Identities = 28/71 (39%), Positives = 40/71 (56%), Gaps = 7/71 (10%)

Query  53   SSSSSHASPRSMSAAAIAGGVLGGIGFTLICSIILALFFRSRRRRQ-----ASRELPLQD  107
            +S+S + SP S+S  AIAG V+G +G  +   IILAL +R RR+R        ++ PL D
Sbjct  252  ASNSGNGSPNSLSRGAIAGIVVGAVGVLIGMGIILAL-WRWRRKRSNKNAGGEKQRPLPD  310

Query  108  NKHAAY-HPEM  117
                 Y  PE+
Sbjct  311  GDGGGYTKPEL  321


>ref|XP_003717394.1| hypothetical protein MGG_10115 [Magnaporthe oryzae 70-15]
 gb|EHA51075.1| hypothetical protein MGG_10115 [Magnaporthe oryzae 70-15]
Length=562

 Score = 45.1 bits (105),  Expect = 0.044, Method: Compositional matrix adjust.
 Identities = 34/91 (37%), Positives = 46/91 (51%), Gaps = 4/91 (4%)

Query  19   SMSTAEGRRTSTYVIYSTSTLAETIEPSSTSTLLSSSSSHASPRSMSAAAIAGGVLGGIG  78
            + S A G      V   T TL   I PSST T  S++S      S SAAA AG   G IG
Sbjct  149  TQSPASGTDAPLAVAKDTPTLP--IAPSSTPTPTSAASKAED--SGSAAAKAGIAFGIIG  204

Query  79   FTLICSIILALFFRSRRRRQASRELPLQDNK  109
              L+ S+++   F+ R+++   REL  +D K
Sbjct  205  GLLVISLLIFFLFKKRKQQMERRELQAEDEK  235


>emb|CRJ79720.1| hypothetical protein BN1708_000082, partial [Verticillium longisporum]
Length=783

 Score = 45.1 bits (105),  Expect = 0.051, Method: Compositional matrix adjust.
 Identities = 19/54 (35%), Positives = 35/54 (65%), Gaps = 0/54 (0%)

Query  52   LSSSSSHASPRSMSAAAIAGGVLGGIGFTLICSIILALFFRSRRRRQASRELPL  105
            L  SS+ +    MSAA IAG V+GG+ F  IC+++   + +++++R+++   PL
Sbjct  339  LPGSSADSESSGMSAAIIAGAVVGGVAFLTICALLFWFWRKNKKKRRSTLLTPL  392


>ref|XP_009656719.1| hypothetical protein VDAG_09463 [Verticillium dahliae VdLs.17]
 gb|EGY19129.1| hypothetical protein VDAG_09463 [Verticillium dahliae VdLs.17]
Length=783

 Score = 45.1 bits (105),  Expect = 0.054, Method: Compositional matrix adjust.
 Identities = 19/54 (35%), Positives = 35/54 (65%), Gaps = 0/54 (0%)

Query  52   LSSSSSHASPRSMSAAAIAGGVLGGIGFTLICSIILALFFRSRRRRQASRELPL  105
            L  SS+ +    MSAA IAG V+GG+ F  IC+++   + +++++R+++   PL
Sbjct  339  LPGSSADSESSGMSAAIIAGAVVGGVAFLTICALLFWFWRKNKKKRRSTLLTPL  392


>emb|CEJ00600.1| hypothetical protein RMCBS344292_14652 [Rhizopus microsporus]
Length=305

 Score = 43.9 bits (102),  Expect = 0.075, Method: Compositional matrix adjust.
 Identities = 24/76 (32%), Positives = 45/76 (59%), Gaps = 8/76 (11%)

Query  28   TSTYVIYSTS-TLAETIEPSSTSTLLSSSSSHASPRSMSAAAIAGGVLGGIGFTLICSII  86
            TST+ + STS ++  T  P++ S ++        P S++++AIAGGV+GG+ F ++  II
Sbjct  154  TSTFTVVSTSYSMYTTSVPTNISDIVK-------PSSVNSSAIAGGVVGGVAFIVLIGII  206

Query  87   LALFFRSRRRRQASRE  102
                 R +  ++ +R+
Sbjct  207  GFFVLRKKNPKKTTRD  222


>ref|XP_008023814.1| hypothetical protein SETTUDRAFT_107372 [Setosphaeria turcica 
Et28A]
 gb|EOA88362.1| hypothetical protein SETTUDRAFT_107372 [Setosphaeria turcica 
Et28A]
Length=396

 Score = 43.9 bits (102),  Expect = 0.10, Method: Compositional matrix adjust.
 Identities = 23/66 (35%), Positives = 37/66 (56%), Gaps = 6/66 (9%)

Query  45   PSSTSTLLSSSSSHASPRSMSAAAIAGGVLGGIGFTLICSIILALFFRSR------RRRQ  98
            P+ST T  ++S    +   +SA AIAG V+GG+ F +IC+ +     RS+      +RR 
Sbjct  202  PTSTGTPPANSGGKGNSHVLSAGAIAGIVVGGVAFLVICAALFFFVGRSKSLKETLKRRD  261

Query  99   ASRELP  104
            A+  +P
Sbjct  262  ATNTMP  267


>gb|EJS42050.1| wsc2p [Saccharomyces arboricola H-6]
Length=500

 Score = 43.5 bits (101),  Expect = 0.14, Method: Compositional matrix adjust.
 Identities = 22/64 (34%), Positives = 38/64 (59%), Gaps = 2/64 (3%)

Query  52   LSSSSSHASPRSMSAAAIAGGVLGGIGFTLICSIILALFFRSRRRRQASRELPLQDNKHA  111
            L+  +S +  + +S  AIAG V+G I  T++   +   FF  ++RRQ+S+ + L++ KH 
Sbjct  301  LNGKTSASKSKGLSGGAIAGVVVGVICGTVLLVALAIFFFIWKKRRQSSQHVDLEETKH-  359

Query  112  AYHP  115
             Y P
Sbjct  360  -YQP  362


>gb|EJT96809.1| hypothetical protein DACRYDRAFT_112426 [Dacryopinax sp. DJM-731 
SS1]
Length=503

 Score = 43.5 bits (101),  Expect = 0.14, Method: Compositional matrix adjust.
 Identities = 27/73 (37%), Positives = 37/73 (51%), Gaps = 4/73 (5%)

Query  63   SMSAAAIAGGVLGGIGFTLICSIILALFFRSRRRRQASRELPLQDNKHAAYHPEM--FMV  120
            S    AI  GV+GGI    +C I+L L+   RRRR A+  L   D      HPE   +++
Sbjct  322  STPVGAIVDGVVGGI--AAVCIIVLILWCFCRRRRHAASALLEIDPDPIFVHPEATPYIL  379

Query  121  PAQASVFGTKSAE  133
            P Q +   T SA+
Sbjct  380  PPQPAQISTNSAQ  392


>gb|KIJ96155.1| hypothetical protein K443DRAFT_682489 [Laccaria amethystina LaAM-08-1]
Length=405

 Score = 42.4 bits (98),  Expect = 0.28, Method: Compositional matrix adjust.
 Identities = 29/92 (32%), Positives = 42/92 (46%), Gaps = 13/92 (14%)

Query  55   SSSHASPRSMSAAAIAGGVLGGIGFTLICSIILALFFRSRRRRQASRELPLQDNKHAA--  112
            SSS A+       AI GGVLGG+    + +II+  +F   R R  ++E+ + D    A  
Sbjct  212  SSSTATHHPAPIGAIVGGVLGGVATIALVTIIVYFYFYRNRDRSTAQEVEVTDQAIFAGP  271

Query  113  YHPEMFMV-----------PAQASVFGTKSAE  133
              P   MV           P+QAS +GT   +
Sbjct  272  QTPRTLMVTPTEIDPFVIPPSQASFYGTAEKQ  303


>emb|CEG64853.1| hypothetical protein RMATCC62417_01760 [Rhizopus microsporus]
Length=222

 Score = 42.0 bits (97),  Expect = 0.31, Method: Compositional matrix adjust.
 Identities = 21/68 (31%), Positives = 40/68 (59%), Gaps = 0/68 (0%)

Query  35   STSTLAETIEPSSTSTLLSSSSSHASPRSMSAAAIAGGVLGGIGFTLICSIILALFFRSR  94
            ST T+  T     T+++ ++ S    P S++++AIAGGV+GG+ F ++  II     R +
Sbjct  72   STFTVVSTSYSMYTTSVPTNISEIQQPSSVNSSAIAGGVVGGVAFIVLIGIIGFFVLRKK  131

Query  95   RRRQASRE  102
              ++ +R+
Sbjct  132  NPKKTTRD  139


>ref|XP_008037507.1| hypothetical protein TRAVEDRAFT_47227 [Trametes versicolor FP-101664 
SS1]
 gb|EIW59930.1| hypothetical protein TRAVEDRAFT_47227 [Trametes versicolor FP-101664 
SS1]
Length=482

 Score = 42.4 bits (98),  Expect = 0.33, Method: Compositional matrix adjust.
 Identities = 23/61 (38%), Positives = 35/61 (57%), Gaps = 0/61 (0%)

Query  42   TIEPSSTSTLLSSSSSHASPRSMSAAAIAGGVLGGIGFTLICSIILALFFRSRRRRQASR  101
            T +PS  + LL + +S     +++A  IAG V+GGI F ++ + IL  F R +R R A  
Sbjct  279  TDDPSEVAGLLRAPTSSTRKSALAAGTIAGSVIGGIVFFVLAAAILWSFIRRKRERLAVF  338

Query  102  E  102
            E
Sbjct  339  E  339


>emb|CEI97351.1| hypothetical protein RMCBS344292_11486 [Rhizopus microsporus]
Length=300

 Score = 42.0 bits (97),  Expect = 0.34, Method: Compositional matrix adjust.
 Identities = 19/61 (31%), Positives = 34/61 (56%), Gaps = 4/61 (7%)

Query  61   PRSMSAAAIAGGVLGGIGFTLICSIILALFFRSRRRRQASRELPLQDNKHAAYHPEMFMV  120
            P S++++AIAGGV+GG+ F ++  II     R +  ++ +R+  +      A  PE +  
Sbjct  176  PSSVNSSAIAGGVVGGVAFIVLIGIIGFFVLRKKNPKKTTRDQEID----VALPPERYGS  231

Query  121  P  121
            P
Sbjct  232  P  232


>emb|CEG64852.1| hypothetical protein RMATCC62417_01760 [Rhizopus microsporus]
Length=300

 Score = 42.0 bits (97),  Expect = 0.36, Method: Compositional matrix adjust.
 Identities = 15/42 (36%), Positives = 28/42 (67%), Gaps = 0/42 (0%)

Query  61   PRSMSAAAIAGGVLGGIGFTLICSIILALFFRSRRRRQASRE  102
            P S++++AIAGGV+GG+ F ++  II     R +  ++ +R+
Sbjct  176  PSSVNSSAIAGGVVGGVAFIVLIGIIGFFVLRKKNPKKTTRD  217


>ref|XP_002552621.1| KLTH0C09196p [Lachancea thermotolerans]
 emb|CAR22183.1| KLTH0C09196p [Lachancea thermotolerans CBS 6340]
Length=439

 Score = 42.0 bits (97),  Expect = 0.43, Method: Compositional matrix adjust.
 Identities = 31/88 (35%), Positives = 51/88 (58%), Gaps = 8/88 (9%)

Query  23   AEGRRTSTYVIYSTSTLAETIEPSSTSTLLSSSSSHA---SPRSMSAAAIAGGVLGGI-G  78
            +   R++ YV  + +T+ E   PSS++   +S+ +++   S +S+S  AIAG V+G I G
Sbjct  278  SNNNRSTMYV--TATTIVEA--PSSSAQANASTKNNSNGKSKKSLSGGAIAGVVIGAIVG  333

Query  79   FTLICSIILALFFRSRRRRQASRELPLQ  106
              LI + +  LF+R R RR+A  E   Q
Sbjct  334  SFLIAATVFFLFWRRRNRREADLEETKQ  361


>gb|KIM94625.1| hypothetical protein OIDMADRAFT_34638 [Oidiodendron maius Zn]
Length=314

 Score = 41.6 bits (96),  Expect = 0.47, Method: Compositional matrix adjust.
 Identities = 22/48 (46%), Positives = 31/48 (65%), Gaps = 0/48 (0%)

Query  49   STLLSSSSSHASPRSMSAAAIAGGVLGGIGFTLICSIILALFFRSRRR  96
            ST  S S +  S   +SA AIAG V+G +   ++ SI+LALF+R RR+
Sbjct  219  STASSGSKAGVSSHGLSAGAIAGIVIGSVAGAILFSILLALFWRRRRQ  266


>gb|KIV87214.1| hypothetical protein PV11_02776 [Exophiala sideris]
Length=539

 Score = 41.6 bits (96),  Expect = 0.51, Method: Compositional matrix adjust.
 Identities = 22/50 (44%), Positives = 32/50 (64%), Gaps = 4/50 (8%)

Query  53   SSSSSHASPRSMSAAAIAGGVLGGI-GFTLICSIILALFFRSRRRRQASR  101
            +SS+SH     +S  AIAG V+G + GF L+C++I+   F  RRRR  S+
Sbjct  255  ASSTSH---HGLSGGAIAGIVVGAVLGFLLLCALIVLCVFCLRRRRHGSK  301


>gb|KIK53882.1| hypothetical protein GYMLUDRAFT_49178 [Gymnopus luxurians FD-317 
M1]
Length=403

 Score = 41.6 bits (96),  Expect = 0.53, Method: Compositional matrix adjust.
 Identities = 23/62 (37%), Positives = 34/62 (55%), Gaps = 8/62 (13%)

Query  67   AAIAGGVLGGIGFTLICSIILALFFRSRRRRQ-ASRELPLQDNKHAAYHPEMFMVPAQAS  125
             AI GG LGG    ++  I L ++ + RR+RQ  +RE      +HA +H +M MV +Q  
Sbjct  197  GAIVGGALGGAVAVMLILIALCIWGKYRRKRQLGNRE------RHATFHGDM-MVKSQVG  249

Query  126  VF  127
             F
Sbjct  250  SF  251


>ref|XP_003666942.1| hypothetical protein MYCTH_2312114 [Myceliophthora thermophila 
ATCC 42464]
 gb|AEO61697.1| hypothetical protein MYCTH_2312114 [Myceliophthora thermophila 
ATCC 42464]
Length=496

 Score = 41.6 bits (96),  Expect = 0.56, Method: Compositional matrix adjust.
 Identities = 26/74 (35%), Positives = 44/74 (59%), Gaps = 6/74 (8%)

Query  32   VIYSTSTLAETIEPSSTSTLLSSSSSHASPRSMSAAAIAGGVLGGIGFTLICSIILALFF  91
             I +++T A+    +++ST   +S++ ++P      AI GGVLGGI    +C I  A+F 
Sbjct  127  TITASTTDADRAGAATSSTPPEASTTQSTP----VGAIVGGVLGGIALIALC-IFGAIFL  181

Query  92   RSRRRR-QASRELP  104
            R +R++ QA  E+P
Sbjct  182  RQQRKKSQAMAEMP  195


>ref|XP_001267184.1| hypothetical protein NFIA_107790 [Neosartorya fischeri NRRL 181]
 gb|EAW25287.1| conserved hypothetical protein [Neosartorya fischeri NRRL 181]
Length=374

 Score = 41.6 bits (96),  Expect = 0.56, Method: Compositional matrix adjust.
 Identities = 28/87 (32%), Positives = 44/87 (51%), Gaps = 2/87 (2%)

Query  39   LAETIEPSSTSTLLSSSSSHASPRSMSAAAIAGGVLGGIGFTLICSIILALFFRSRRRRQ  98
             +E++ P ST  L+S S+   S  S  A  IA  +LG +   LIC + +  FF  R RR+
Sbjct  208  FSESLLPQSTVDLISPSAKPNSDSSRLALIIALPILGFV--ILICILAVGCFFFIRWRRK  265

Query  99   ASRELPLQDNKHAAYHPEMFMVPAQAS  125
             +R+    D+ HA ++      PA  +
Sbjct  266  QARKNRRSDHLHARWNDTTISTPAHGA  292


>ref|XP_005996350.1| PREDICTED: ephrin type-A receptor 2 [Latimeria chalumnae]
Length=979

 Score = 41.6 bits (96),  Expect = 0.69, Method: Compositional matrix adjust.
 Identities = 24/76 (32%), Positives = 39/76 (51%), Gaps = 1/76 (1%)

Query  29   STYVIYSTSTLAETIEPSSTSTLLSSSSSHASPRSMSAAAIAGGVLGGIGFTLICSIILA  88
            +TYV    +  +E +  +S     ++ SS A P  +S  A+ GG + G    ++  I   
Sbjct  500  TTYVFRIQALTSEGVGGNSMEQEFTTLSSDADPEGVSKTAVIGGAVAG-SIIILLLIFAV  558

Query  89   LFFRSRRRRQASRELP  104
            LFFR R+RR  +R+ P
Sbjct  559  LFFRHRQRRSHARQGP  574


>gb|KFH43510.1| hypothetical protein ACRE_057260 [Acremonium chrysogenum ATCC 
11550]
Length=447

 Score = 41.2 bits (95),  Expect = 0.69, Method: Compositional matrix adjust.
 Identities = 30/91 (33%), Positives = 44/91 (48%), Gaps = 9/91 (10%)

Query  27   RTSTYVIYSTSTLAETIEPSSTSTLLSSSSSHA-----SPRSMSAAAIAGGVLGGIGFTL  81
            RT T  + +T     T EPS+T        + A     S R  +  AI GG LGGI F +
Sbjct  221  RTRTRELSTTVEETSTSEPSATGVDGDEGGASAPETSDSDRPFNVGAIVGGTLGGIAFMV  280

Query  82   ICSIILALFFRSRRRRQASRELPLQ--DNKH  110
              +++  +F+  RR +  SR+ P Q  D +H
Sbjct  281  SAAVL--MFYLWRRNKGNSRQGPGQMSDTQH  309


>emb|CCA70236.1| hypothetical protein PIIN_04175 [Piriformospora indica DSM 11827]
Length=600

 Score = 41.2 bits (95),  Expect = 0.70, Method: Compositional matrix adjust.
 Identities = 17/40 (43%), Positives = 23/40 (58%), Gaps = 0/40 (0%)

Query  67   AAIAGGVLGGIGFTLICSIILALFFRSRRRRQASRELPLQ  106
             AIAGGVLGG+ F  I  + +  F R +RR +  R  P +
Sbjct  390  GAIAGGVLGGLAFLTIVIVGILFFLRHKRREEEKRRAPTE  429


>gb|KFY41578.1| hypothetical protein V494_02916 [Pseudogymnoascus pannorum VKM 
F-4513 (FW-928)]
Length=548

 Score = 41.2 bits (95),  Expect = 0.81, Method: Compositional matrix adjust.
 Identities = 38/126 (30%), Positives = 62/126 (49%), Gaps = 14/126 (11%)

Query  46   SSTSTLLSSSSSHASPRSMSAAAIAGGVLGGIGFTLICSIILALFFRSRRRRQASRELPL  105
            SST+T+ +S+ S      +S  AIAG  +G +   +I +I+  L +R +R+R   R+L L
Sbjct  197  SSTNTVSTSNLSSEKKSGLSTGAIAGTAVGVVCILIIVAIVFFLCYRRQRKR---RDL-L  252

Query  106  QDNKHAAYHPEMFMVPAQASVFGTKSAEVNGEKTLRSIETGGHARTPSEVP--LLTEPPL  163
            Q    A+Y P    +  +AS     S+ +NG+      +T G   T  + P  + T  P 
Sbjct  253  QHAGDASYDPRNSNMSQKAS---DPSSHINGDP-----DTPGQNYTEPKSPGYVATSNPN  304

Query  164  APMPYS  169
             P  Y+
Sbjct  305  GPDDYN  310


>ref|XP_006453939.1| hypothetical protein AGABI2DRAFT_175677 [Agaricus bisporus var. 
bisporus H97]
 gb|EKV50953.1| hypothetical protein AGABI2DRAFT_175677 [Agaricus bisporus var. 
bisporus H97]
Length=418

 Score = 40.8 bits (94),  Expect = 0.86, Method: Compositional matrix adjust.
 Identities = 21/51 (41%), Positives = 32/51 (63%), Gaps = 2/51 (4%)

Query  54   SSSSHASPRSMSAAAIAGGVLGGIGFTLICSIILALFFRSRRRRQASRELP  104
            + S+  +P++ +AA IAGGV+GGI   L+ + I    F  RRRR+A   +P
Sbjct  229  TGSTLVTPKTSNAAMIAGGVVGGIMGLLVLAAIFV--FLHRRRRKAENVMP  277


>gb|KIK95982.1| hypothetical protein PAXRUDRAFT_139503 [Paxillus rubicundulus 
Ve08.2h10]
Length=402

 Score = 40.4 bits (93),  Expect = 1.2, Method: Compositional matrix adjust.
 Identities = 20/35 (57%), Positives = 26/35 (74%), Gaps = 1/35 (3%)

Query  67   AAIAGGVLGGIGFTLICSIILALFFRSRRRRQASR  101
             AIAGGVLGG+   ++  + LALF R+ RRRQAS+
Sbjct  176  GAIAGGVLGGLVIIMVL-VGLALFLRALRRRQASK  209


>ref|XP_003030568.1| expressed protein [Schizophyllum commune H4-8]
 gb|EFI95665.1| expressed protein [Schizophyllum commune H4-8]
Length=210

 Score = 39.7 bits (91),  Expect = 1.3, Method: Compositional matrix adjust.
 Identities = 23/52 (44%), Positives = 29/52 (56%), Gaps = 2/52 (4%)

Query  53   SSSSSHASPRSMSAAAIAGGVLGGIGFTLICSIILALFFRSRR--RRQASRE  102
            S++ +HAS   +S  AIAG V+G I  T I + IL L  R R   RR   RE
Sbjct  125  SNNQTHASKHKLSGGAIAGIVIGSIAGTAILAFILWLLCRRRWNTRRYGDRE  176


>gb|KIM55198.1| hypothetical protein SCLCIDRAFT_1221343 [Scleroderma citrinum 
Foug A]
Length=428

 Score = 40.4 bits (93),  Expect = 1.3, Method: Compositional matrix adjust.
 Identities = 26/60 (43%), Positives = 31/60 (52%), Gaps = 7/60 (12%)

Query  61   PRSMSAAAIAGGVLGGIGFTLICSIILALFFRSRRRRQASREL-----PLQDNKHAAYHP  115
            P       I GGVLGGI   LI S+I AL FR R++RQ   +L     P   + HA Y P
Sbjct  205  PSHSDTGTIVGGVLGGIALVLI-SVIAALCFR-RKKRQNKIDLSTDADPRPFDPHALYDP  262


>dbj|GAO83721.1| mediator of RNA polymerase II transcription subunit 8 [Neosartorya 
udagawae]
Length=595

 Score = 40.4 bits (93),  Expect = 1.3, Method: Compositional matrix adjust.
 Identities = 28/88 (32%), Positives = 43/88 (49%), Gaps = 2/88 (2%)

Query  38   TLAETIEPSSTSTLLSSSSSHASPRSMSAAAIAGGVLGGIGFTLICSIILALFFRSRRRR  97
              +E++ P ST  L S S +  S  S  A  IA  +LG +   LIC + +  FF  R RR
Sbjct  217  VFSESLLPQSTVDLTSPSGNANSGSSRLALIIALPILGFV--ILICILAVGCFFFIRWRR  274

Query  98   QASRELPLQDNKHAAYHPEMFMVPAQAS  125
            + +R+    D+ HA ++      PA  +
Sbjct  275  KQARKHRRSDHLHARWNDTTISTPAHGA  302


>ref|XP_011123584.1| hypothetical protein AOL_s00083g103 [Arthrobotrys oligospora 
ATCC 24927]
 gb|EGX47595.1| hypothetical protein AOL_s00083g103 [Arthrobotrys oligospora 
ATCC 24927]
Length=430

 Score = 40.0 bits (92),  Expect = 1.5, Method: Compositional matrix adjust.
 Identities = 25/57 (44%), Positives = 32/57 (56%), Gaps = 3/57 (5%)

Query  67   AAIAGGVLGGIGFTLICSIILALFFRSR-RRRQASRELPLQDNKHAAYHPEMFMVPA  122
            AAIAG V+GGIG   I  +I+AL FR R  R  ++   P Q N H    P M + P+
Sbjct  207  AAIAGIVVGGIGAMAIIGLIIALVFRYRVNRSDSTASNPPQHNSH--LQPRMGLAPS  261


>gb|KNE72351.1| hypothetical protein AMAG_16835 [Allomyces macrogynus ATCC 38327]
Length=681

 Score = 39.7 bits (91),  Expect = 2.3, Method: Compositional matrix adjust.
 Identities = 20/47 (43%), Positives = 31/47 (66%), Gaps = 1/47 (2%)

Query  63   SMSAAAIAGGVLGGIGFTLICSIILALFFRSRRRRQASRELPLQDNK  109
            S S AA+ GG+LGG+   L+ +I  ALF   RRR++A+++  L D +
Sbjct  264  SSSNAALIGGILGGV-VILLAAIGAALFLVHRRRQRAAKQQQLDDKQ  309


>ref|XP_008726262.1| hypothetical protein G647_03695 [Cladophialophora carrionii CBS 
160.54]
 gb|ETI24326.1| hypothetical protein G647_03695 [Cladophialophora carrionii CBS 
160.54]
Length=528

 Score = 39.3 bits (90),  Expect = 2.9, Method: Compositional matrix adjust.
 Identities = 17/37 (46%), Positives = 25/37 (68%), Gaps = 2/37 (5%)

Query  65   SAAAIAGGVLGGIGFTLICSIILALFFRSRRRRQASR  101
            +A  IAGGV+GG+   LI +I+  ++F  RRR+Q S 
Sbjct  428  NAGPIAGGVVGGVA--LIAAILFGIWFCQRRRKQRSH  462


>ref|WP_049673322.1| hypothetical protein [Desulfocarbo indianensis]
 gb|KMY68362.1| hypothetical protein AAU61_01320 [Desulfocarbo indianensis]
Length=789

 Score = 39.3 bits (90),  Expect = 3.5, Method: Compositional matrix adjust.
 Identities = 31/125 (25%), Positives = 54/125 (43%), Gaps = 0/125 (0%)

Query  62   RSMSAAAIAGGVLGGIGFTLICSIILALFFRSRRRRQASRELPLQDNKHAAYHPEMFMVP  121
            R +    +A G+L     T   SI+L L +++  RR+  ++  +     AA    ++MV 
Sbjct  128  RELGNGFLAAGLLFLGALTFKTSIVLRLLWKAHLRRRPGQDDAMSFRGQAAVFVALWMVL  187

Query  122  AQASVFGTKSAEVNGEKTLRSIETGGHARTPSEVPLLTEPPLAPMPYSVPPISTMAPARL  181
              A+V+  ++    GE+    +E   + R P  V    +  LA   +  P     AP R+
Sbjct  188  GLAAVWVNQAVSTTGEEAGLLLEAEAYRRGPGFVASQEDQRLAAKEFHWPLEEGQAPPRM  247

Query  182  IQGSV  186
              G V
Sbjct  248  KSGGV  252


>ref|XP_010869136.1| PREDICTED: transmembrane protein 248 isoform X1 [Esox lucius]
Length=336

 Score = 38.9 bits (89),  Expect = 3.8, Method: Compositional matrix adjust.
 Identities = 38/137 (28%), Positives = 58/137 (42%), Gaps = 30/137 (22%)

Query  134  VNGEKTLRSIETGGHARTPSEVPLLTEPPLAPMPYSVPPISTMAPAR-------------  180
            +N   T  S  T G AR+ ++ PLL E P  P+  SVP   T+ P R             
Sbjct  100  LNESTTPESTATSGQARSSTQAPLLLEDP-GPINISVPITLTLDPLRPFGGYSRNITHLY  158

Query  181  --LIQGSVSLSSLRPSRSEQSIYPLLSSSASSDELM-QAHDPFGAYA------------P  225
              ++ G V L+  R +  E +I   L +S +SDE M   H     ++            P
Sbjct  159  ATVLAGQVGLAG-REAHEEMNITFTLPASWNSDECMLHGHCEQVVFSSCMTITAASNVFP  217

Query  226  VSIIPPRSIPRSSSRTT  242
            V++ PP  +P + +  T
Sbjct  218  VTVQPPHCVPETYTNAT  234


>ref|XP_010869137.1| PREDICTED: transmembrane protein 248 isoform X2 [Esox lucius]
Length=316

 Score = 38.9 bits (89),  Expect = 4.0, Method: Compositional matrix adjust.
 Identities = 38/137 (28%), Positives = 58/137 (42%), Gaps = 30/137 (22%)

Query  134  VNGEKTLRSIETGGHARTPSEVPLLTEPPLAPMPYSVPPISTMAPAR-------------  180
            +N   T  S  T G AR+ ++ PLL E P  P+  SVP   T+ P R             
Sbjct  80   LNESTTPESTATSGQARSSTQAPLLLEDP-GPINISVPITLTLDPLRPFGGYSRNITHLY  138

Query  181  --LIQGSVSLSSLRPSRSEQSIYPLLSSSASSDELM-QAHDPFGAYA------------P  225
              ++ G V L+  R +  E +I   L +S +SDE M   H     ++            P
Sbjct  139  ATVLAGQVGLAG-REAHEEMNITFTLPASWNSDECMLHGHCEQVVFSSCMTITAASNVFP  197

Query  226  VSIIPPRSIPRSSSRTT  242
            V++ PP  +P + +  T
Sbjct  198  VTVQPPHCVPETYTNAT  214


>emb|CBY12936.1| unnamed protein product [Oikopleura dioica]
Length=640

 Score = 38.9 bits (89),  Expect = 4.1, Method: Compositional matrix adjust.
 Identities = 32/109 (29%), Positives = 53/109 (49%), Gaps = 23/109 (21%)

Query  15   DAEGSMSTAEGRRTSTYVIYSTSTL--AETIEPSSTSTLL-SSSSSHASPRSMSAAAIAG  71
            D E +++ A G  T+   +YSTST+   E++     + L  S SS++ + R  S    AG
Sbjct  398  DTEFTVTPASGEVTT---LYSTSTIRAVESVTSGPNAVLNPSQSSANTNSRPPSVIETAG  454

Query  72   GVLG-----------------GIGFTLICSIILALFFRSRRRRQASREL  103
              +G                  IG TL+C+I+L +F++ RRR+ +  E 
Sbjct  455  EPIGPVTTGEPLRRTRIVIGLFIGLTLVCAIVLIIFYKKRRRKPSQNEW  503


>gb|EKG09317.1| hypothetical protein MPH_13631 [Macrophomina phaseolina MS6]
Length=693

 Score = 38.9 bits (89),  Expect = 4.1, Method: Compositional matrix adjust.
 Identities = 15/43 (35%), Positives = 27/43 (63%), Gaps = 2/43 (5%)

Query  69   IAGGVLGGIGFTLICSIILALF--FRSRRRRQASRELPLQDNK  109
            ++GG   G+ F +IC I L +F  F  RR++Q  +++ +Q+ K
Sbjct  175  MSGGAKAGMAFAIICFIALVVFLAFFWRRKQQEKKQMAVQNEK  217


>emb|CBY43440.1| unnamed protein product [Oikopleura dioica]
Length=372

 Score = 38.5 bits (88),  Expect = 4.6, Method: Compositional matrix adjust.
 Identities = 32/109 (29%), Positives = 53/109 (49%), Gaps = 23/109 (21%)

Query  15   DAEGSMSTAEGRRTSTYVIYSTSTL--AETIEPSSTSTLL-SSSSSHASPRSMSAAAIAG  71
            D E +++ A G  T+   +YSTST+   E++     + L  S SS++ + R  S    AG
Sbjct  78   DTEFTVTPASGEVTT---LYSTSTIRAVESVTSGPNAVLNPSQSSANTNSRPPSVIETAG  134

Query  72   GVLG-----------------GIGFTLICSIILALFFRSRRRRQASREL  103
              +G                  IG TL+C+I+L +F++ RRR+ +  E 
Sbjct  135  KPIGPVTTGEPLRRTRIVIGLFIGLTLVCAIVLIIFYKKRRRKPSQNEW  183


>gb|KIK55984.1| hypothetical protein GYMLUDRAFT_248203 [Gymnopus luxurians FD-317 
M1]
Length=345

 Score = 38.5 bits (88),  Expect = 4.7, Method: Compositional matrix adjust.
 Identities = 24/47 (51%), Positives = 28/47 (60%), Gaps = 3/47 (6%)

Query  55   SSSHASPRS---MSAAAIAGGVLGGIGFTLICSIILALFFRSRRRRQ  98
            SS  ASP+S   +SAAAIAG V+G I   +I S  L  F R RR R 
Sbjct  168  SSGVASPKSHKAVSAAAIAGSVVGSIVALIIASFCLFCFLRRRRARN  214


>gb|KIL85067.1| hypothetical protein FAVG1_11495 [Fusarium avenaceum]
Length=224

 Score = 38.1 bits (87),  Expect = 4.7, Method: Compositional matrix adjust.
 Identities = 24/78 (31%), Positives = 43/78 (55%), Gaps = 6/78 (8%)

Query  41   ETIEPSSTSTLLSSSSS--HASPRSMSAAAIAGGVLGGI-GFTLICSIILALFFRSRRRR  97
            E+  P+  S L +S++S   +SP+ ++   IAG V+G + G   I S+I  L    +RR+
Sbjct  118  ESESPTGESNLPTSATSSPESSPQGLTTGGIAGVVIGAVAGGVAIASLIFWLL---KRRK  174

Query  98   QASRELPLQDNKHAAYHP  115
            +   + P+Q   +  Y+P
Sbjct  175  RTDNQAPMQPTSYEYYNP  192


>gb|KIY49433.1| hypothetical protein FISHEDRAFT_72756 [Fistulina hepatica ATCC 
64428]
Length=437

 Score = 38.5 bits (88),  Expect = 5.5, Method: Compositional matrix adjust.
 Identities = 32/92 (35%), Positives = 48/92 (52%), Gaps = 6/92 (7%)

Query  42   TIEPSSTSTLLSSSSSHASPRSMSAAAIAGGVLGGIGFTLICSIILALFFRSRRRRQASR  101
            T+ PS+ +    +SS + S +  + AAI GG +GGI F  + + IL L    RR+RQA+ 
Sbjct  305  TLAPSAHNNTSEASSQNRSSKD-NVAAIVGGTVGGIVFVALVATILFLLL--RRQRQAAM  361

Query  102  ELPLQDNKHAA--YHPEMFMVPAQASVFGTKS  131
                +  +HA   Y  + F  P + S  GT S
Sbjct  362  TYRNEMAEHALTPYQQQAFDRPLK-SAMGTPS  392


>gb|EQL31746.1| hypothetical protein, variant 3 [Blastomyces dermatitidis ATCC 
26199]
Length=214

 Score = 37.7 bits (86),  Expect = 7.2, Method: Compositional matrix adjust.
 Identities = 21/54 (39%), Positives = 30/54 (56%), Gaps = 1/54 (2%)

Query  63   SMSAAAIAGGVLGGI-GFTLICSIILALFFRSRRRRQASRELPLQDNKHAAYHP  115
            S+S  AIAG V+G + G  +I S IL LF+  RRR +   ++ +    H A  P
Sbjct  84   SLSGGAIAGIVVGTLLGIGVIVSAILWLFYIRRRREREDSKVGMNGYDHPASSP  137


>ref|XP_007775861.1| hypothetical protein CONPUDRAFT_86099 [Coniophora puteana RWD-64-598 
SS2]
 gb|EIW73960.1| hypothetical protein CONPUDRAFT_86099 [Coniophora puteana RWD-64-598 
SS2]
Length=831

 Score = 38.1 bits (87),  Expect = 7.2, Method: Compositional matrix adjust.
 Identities = 18/43 (42%), Positives = 24/43 (56%), Gaps = 0/43 (0%)

Query  64   MSAAAIAGGVLGGIGFTLICSIILALFFRSRRRRQASRELPLQ  106
            +S  AI G ++GGI F LI   +L  + R R RRQ  R+  L 
Sbjct  113  LSKGAIVGIIVGGIFFALILLTLLICYARWRNRRQHGRQRGLN  155


>ref|XP_001796011.1| hypothetical protein SNOG_05608 [Phaeosphaeria nodorum SN15]
 gb|EAT86672.1| hypothetical protein SNOG_05608 [Phaeosphaeria nodorum SN15]
Length=170

 Score = 37.4 bits (85),  Expect = 7.3, Method: Compositional matrix adjust.
 Identities = 27/79 (34%), Positives = 40/79 (51%), Gaps = 2/79 (3%)

Query  29   STYVIYSTSTLAETIEPSSTSTLLSSSSSHASPRSMSAAAIAGGVLGGIGFTLICSIILA  88
            S YVI   +        SS  T   +S+S  S  S+S   IAG V+G I   ++  I+ +
Sbjct  21   SPYVIGGPTVAFVAARTSSHDTAAGASTSSQSRNSLSGGTIAGIVVGSIAALVV--ILSS  78

Query  89   LFFRSRRRRQASRELPLQD  107
            +F R  R+R  + +L LQD
Sbjct  79   IFLRWCRKRNQASDLALQD  97


>gb|KMW67547.1| ER membrane protein Wsc4, variant 4 [Blastomyces dermatitidis 
ATCC 18188]
Length=214

 Score = 37.4 bits (85),  Expect = 7.7, Method: Compositional matrix adjust.
 Identities = 21/54 (39%), Positives = 30/54 (56%), Gaps = 1/54 (2%)

Query  63   SMSAAAIAGGVLGGI-GFTLICSIILALFFRSRRRRQASRELPLQDNKHAAYHP  115
            S+S  AIAG V+G + G  +I S IL LF+  RRR +   ++ +    H A  P
Sbjct  84   SLSGGAIAGIVVGTLLGIGVIVSAILWLFYIRRRREREDSKVGMNGYDHPASSP  137


>gb|KJA24276.1| hypothetical protein HYPSUDRAFT_136642 [Hypholoma sublateritium 
FD-334 SS-4]
Length=468

 Score = 38.1 bits (87),  Expect = 7.7, Method: Compositional matrix adjust.
 Identities = 40/117 (34%), Positives = 59/117 (50%), Gaps = 17/117 (15%)

Query  46   SSTSTLLSSSSSHASPRSMSAAAIAGGVLGGIGFTLICSIILALFFRSRRRRQASRELPL  105
            +S+S  L+S++S AS +S +   I G V+GG+   LI   ILA+FF  RR ++   E  L
Sbjct  291  TSSSPALTSNNS-ASGKS-NTGIIVGAVIGGL--VLIALTILAVFFIRRRDKKLRHEEKL  346

Query  106  QDNKHAAYHPEMFM---VPAQASVF-GTKSAEV-----NGEKTLRSIETGGHARTPS  153
                 + + P+ FM   VP   SV  G +S  V        +    I  GGH++ PS
Sbjct  347  P----STFEPQPFMYSPVPITTSVLDGYRSGAVLPSSKQPARGAEGIGYGGHSQMPS  399


Lambda      K        H        a         alpha
   0.315    0.127    0.353    0.792     4.96 

Gapped
Lambda      K        H        a         alpha    sigma
   0.267   0.0410    0.140     1.90     42.6     43.6 

Effective search space used: 1560719411500


  Database: nr
    Posted date:  Sep 23, 2015 12:05 AM
  Number of letters in database: 26,053,659,533
  Number of sequences in database:  71,551,133


Matrix: BLOSUM62
Gap Penalties: Existence: 11, Extension: 1
Neighboring words threshold: 11
Window for multiple hits: 40
```
